# Linaclotide for the treatment of refractory lower bowel manifestations of systemic sclerosis

**DOI:** 10.1186/s12876-021-01738-0

**Published:** 2021-04-15

**Authors:** Eric J. Dein, Fredrick M. Wigley, Zsuzsanna H. McMahan

**Affiliations:** Division of Rheumatology, Johns Hopkins University School of Medicine, 5200 Eastern Avenue, Suite 5200, Mason F. Lord Building, Center Tower, Baltimore, MD 21224 USA

**Keywords:** Scleroderma, Systemic sclerosis, Gastrointestinal, Constipation, Linaclotide, Pseudo-obstruction, Pro-motility, Dysmotility, Clinical

## Abstract

**Background:**

Lower gastrointestinal (GI) tract involvement can affect up to 50% of systemic sclerosis (SSc) patients, and may result in malabsorption, pseudo-obstruction, hospitalization, and death. We report our experience with linaclotide, a selective agonist of guanylate cyclase C (GC-C), for SSc patients with refractory lower GI disease.

**Methods:**

We performed an analysis of patients seen at the Johns Hopkins Scleroderma Center and identified patients prescribed linaclotide for refractory lower GI manifestations. Patients had clinical data collected in our longitudinal database. Linaclotide responders were on medication for at least 12 months with documented effectiveness by the treating physician.

**Results:**

Thirty-one patients with SSc were treated with linaclotide. At the time of linaclotide initiation, 23 of these patients (74%) were classified as having severe GI disease, as defined by recurrent pseudo-obstruction, malabsorption, and/or need for artificial nutrition (Medsger GI severity score ≥ 3). The majority of patients (90.3%; 28/31) had a treatment response, while only three patients (9.7%) reported ineffectiveness or intolerable side effects. Low-dose linaclotide (≤ 145 mcg daily) was used in 18 patients and was effective in 94%. High-dose therapy (> 145 mcg daily) was effective in 11 of 13 patients (85%). Common side effects were diarrhea, cramping, or bloating (11/31, 35%). Ineffectiveness, cost, and abdominal pain were complaints cited among those who discontinued therapy.

**Conclusion:**

Linaclotide is a well-tolerated and efficacious pro-secretory and pro-motility agent that can be used to manage refractory lower GI manifestations in SSc. We found that low-dose linaclotide is an effective option and may be better tolerated, though a subset of patients may require high dose regimens.

## Introduction

Up to 90% of patients with systemic sclerosis (SSc) have gastrointestinal complications [1]. Lower gastrointestinal tract involvement can affect nearly 50% of SSc patients, and may result in malabsorption, recurrent pseudo-obstruction, hospitalization, and death [1–4]. A number of promotility and prosecretory agents exist for colonic dysmotility, but their usage is often limited by adverse effects or limited evidence in SSc [1, 5, 6].

Linaclotide is a selective agonist of guanylate cyclase C (GC-C) and has been demonstrated to be a safe, effective prosecretory and promotility option for refractory constipation in other conditions. The activation of cyclic guanosine monophosphate (cGMP) by GC-C on intestinal epithelial cells increases intestinal secretion and gut motility through nerve activity and fluid homeostasis [7, 8]. Linaclotide for treatment of constipation demonstrated efficacy in multiple randomized, double-blind, placebo-controlled multicenter clinical trials, and across several different disease processes [9–12]. To date, however, no studies have evaluated linaclotide in patients with autoimmune disorders, including SSc.

In this study, we report our experience with linaclotide’s tolerability and benefit in SSc patients with severe, refractory GI dysmotility, recurrent pseudo-obstruction and/or significant constipation who do not respond adequately to first line agents. We aim to provide rheumatologists, who do not routinely use this class of medications, with another therapeutic option for managing SSc-related lower bowel complications in patients with more severe disease.

## Methods

### Study Population

All patients were seen at the Johns Hopkins Scleroderma Center as part of routine clinical care between August 2012 and January 2019. Patients with SSc who were prescribed linaclotide during their time in our Center were identified in our clinical database for retrospective study. Inclusion criteria for subsequent chart review otherwise required age greater than or equal to 18 years old and a diagnosis of SSc based on 2013 ACR/EULAR criteria, 1980 American College of Rheumatology (ACR) criteria or satisfying at least three of five CREST criteria [13].

### Study design, outcome measures, and data abstraction

This study was a restrospective case series reporting our Center’s experience with linaclotide in SSc patients with severe GI complications. All patients were required to report symptoms of constipation (e.g. ≤ 3 bowel movements per week), despite the use of at least one other medication (e.g. polyethelene glycol, senna). Symptom response was defined by an improvement in the number of bowel movements, stool consistency, ease of evacuation, and/or associated symptoms (abdominal pain, distention, bloating, etc.) in the absence of intolerable side effects documented in the medical record, and/or the continuation of treatment for at least 12 months.

All included patients were required to have at least one follow-up visit or telephone note documenting compliance with the medication for at least two weeks. Patients were excluded from the analysis if they did not take the prescribed the medication, or lacked follow-up at least two weeks after the index visit. Patients still receiving treatment or who stopped the medication due to cost concerns were included as responders regardless of length of treatment if they had documentation of symptom improvement. Patients were classified as non-responders if they stopped the medication within the first 12 months due to lack of response or intolerable side effects. As clinical practice varied on dosing, we also classified patients as high or low dose regimens for comparison. High dose linaclotide was defined by a dose of greater than 145 mcg daily. Low dose linaclotide included patients with 145 mcg daily dosing or less, including as needed regimens.

Once identified as having taken linaclotide, the patients’ charts were reviewed to screen for inclusion and exclusion criteria and to collect additional details relevant to the study. The date of first linaclotide prescription was designated as the index visit. All subsequent outpatient notes and telephone calls were reviewed for documentation of medication adherence or discontinuation, dose modification, response to treatment, and reported adverse effects. Data abstraction was completed using a standardized data abstraction sheet so that all charts were systematically reviewed for the same information.

### Clinical phenotyping in the Johns Hopkins scleroderma research registry

All patients were part of the Johns Hopkins Scleroderma Research Registry. In this registry, clinical data is collected at baseline and every six months on all actively followed patients. The registry includes demographic and clinical data, such as age at first clinical visit, sex, race, disease duration, SSc skin subtype, and autoantibody status. Specific organ involvement is defined by physician-determined Medsger severity scores and other objective measures (blood work, imaging, pulmonary function tests, etc.) [2]. To measure patient-reported GI symptoms, we also collect the University of California, Los Angeles Gastrointestinal Tract Instrument (UCLA GIT 2.0) questionnaire [14]. Autoantibody data is systematically obtained on all enrolled patients using the commercially available line immunoblot assay (Systemic Sclerosis Profile Euroline [IgG]; Euroimmun, Lubeck, Germany). This registry data was coupled with the data abstracted from the retrospective chart review to develop the comprehensive dataset.

### Statistical analysis

Chi square and Fishers exact tests were used to compare dichotomous variables. Student’s t-tests were used to assess differences between parametric continuous variables in low and high-dose groups. Non-parametric continuous variables were evaluated with the Wilcoxon-Mann–Whitney test. All statistical analyses were performed using Stata, version 14.2 (StataCorp, College Station, TX).

Written informed consent was obtained on all patients. The present study was approved by the Johns Hopkins Institutional Review Board.

## Results

### Patient attributes

We identified 38 candidate study subjects within the Johns Hopkins Scleroderma Center Research Registry who were prescribed linaclotide. Seven patients were excluded from our analysis: 1 patient did not meet SSc criteria, 2 patients initiated therapy prior to their initial evaluation in our center, and 4 lacked appropriate follow-up after initiating linaclotide. All patients included in our analysis had follow-up confirming patient-reported compliance with linaclotide, with a mean of 7 outpatient clinic visits with rheumatology or gastroenterology (range 0–36) while on linaclotide, and a mean of 1 telephone or electronic message about linaclotide usage (range 0–7), for a total mean of 8 physician contacts (range 1–36).

The 31 patients included in our study had a mean age of 52 years (± 11 years). They were predominantly female (29/31 patients, 94%) and Caucasian (21/31, 68%). Median disease duration (from earliest symptom—Raynaud’s or non-Raynaud’s) was 9 years (IQR: 5–14 years). Commonly affected extra-intestinal organs included Sicca symptoms (26/31, 84%), lung involvement (Medgser score > 1) (22/31, 71%), and Raynaud’s phenomenon (19/31, 61%). Antibodies present among these patients included anti-Scl-70 (7/31, 23%) and anti-CENP (7/31, 23%). Additional clinical features, including extra-intestinal manifestations, the presence of other autoantibodies, and medications used prior to linaclotide are show in Table 1. Of note, polyethylene glycol (22/31, 71%), docusate (13/31, 42%) and senna (10/31, 32%) were the most commonly used medications for constipation prior to linaclotide. As combination therapy was permitted with linaclotide, Table 2 documents medications that were continued while on linaclotide, as well as new medications initiated while on linaclotide. Polyethylene glycol (17/31, 55%), oral docusate (8/31, 26%) and probiotics (8/31, 26%) were the most common medications continued while on linaclotide, while pyridostigmine (8/31, 26%), senna (5/31, 16%), and polyethylene glycol (4/31, 13%) were initiated while on linaclotide therapy.

**Table 1:** Patient demographics

Clinical variable	n = 31
Age at first linaclotide dose, mean (SD)	52 (11)
Female sex, % (n)	94 (29)
White race, % (n)	68 (21)
SSc type: Diffuse cutaneous disease, % (n)	32 (10)
Disease duration in years, RP or non-RP, median (IQR)	9 (5–14)
Medsger GI score at initiation of linaclotide, % (n)	
0 (Normal)	0 (0)
1 (Requiring medications for reflux or abnormal small bowel series)	6 (2)
2 (High-dose reflux medications or antibiotics for bacterial overgrowth)	19 (6)
3 (Malabsorption syndrome or episodes of pseudo-obstruction)	65 (20)
4 (Requiring total parental nutrition)	10 (3)
Sicca symptoms, % (n)	84 (26)
Myopathy, % (n)	42 (13)
Cardiac involvement (Medsger ≥ 1), % (n)	55 (17)
Lung involvement (Medsger > 1), % (n)	71 (22)
Raynaud’s (Medsger ≥ 2), % (n)	61 (19)
Antibody status	
Anti-Scl-70 antibodies, % (n)	23 (7)
Anti-CENP antibodies, % (n)	23 (7)
Anti-RNA pol-3 antibodies, % (n)	10 (3)
Anti-U3RNP antibodies, % (n)	13 (4)
UCLA GIT 2.0 constipation score, mean (SD) n = 9	1.03 (0.55)
Whole gut scintigraphy data	
Abnormal large bowel transit at 72 h, % (n)	70 (7/10)
Percent large bowel emptying at 72 h, median (IQR)	9.5 (0–84)
Medications used prior to linaclotide	
Polyethylene glycol, % (n)	71 (22)
Senna, % (n)	32 (10)
Oral docusate, % (n)	42 (13)
Docusate suppository, % (n)	13 (4)
Tegaserod, % (n)	10 (3)
Lubiprostone, % (n)	29 (9)
Pyridostigmine, % (n)	13 (4)
Prucalopride, % (n)	3 (1)

UCLA GIT (None-to-mild: 0.00–0.49); Whole gut scintigraphy (Normal % colonic emptying at 72 h ≥ 67%)

**Table 2:** Co-medications used with linaclotide

	Prior medications continued with linaclotide (%)	Co-medications initiated while on linaclotide (%)
Polyethylene glycol	17 (55%)	4 (13%)
Senna	5 (16%)	5 (16%)
Oral docusate	8 (26%)	2 (6%)
Docusate suppository	2 (6%)	2 (6%)
Lubiprostone	3 (10%)	0
Pyridostigmine	3 (10%)	8 (26%)
Prucalopride	1 (3%)	0
Domperidone	3 (10%)	0
Erythromycin	0	2 (6%)
Metoclopramide	5 (16%)	1 (3%)
Probiotics	8 (26%)	2 (6%)
Methylnaltrexone	0	3 (10%)

The majority of patients in our study had severe lower GI disease. At the time of linaclotide initiation, 23 patients (74%) were classified as having severe GI disease by their treating physician by the Medsger GI severity score [severe score ≥ 3 denotes a history of recurrent pseudo-obstruction, malabsorption, or need for artificial nutrition] [2]. Ten patients also had whole gut scintigraphy studies, with 70% (7/10) of these patients having severely abnormal colonic transit [median percent colonic emptying of only 9.5% at 72 h (normal ≥ 67%)]. Eight patients had anorectal manometry, with abnormal findings in 7 patients (87.5%). Three patients had dyssenergy and four had hypotensive squeeze. Nine patients had UCLA GIT scores completed prior to linaclotide initiation and had scores in the severe range (1.01–3.00), with a mean constipation domain score of 1.03 (SD: 0.55).

### Symptom response and dosing

Patients in our study were on therapy for a mean duration of 22.6 months (SD ± 18 months) and 90% (28/31) of patients responded to treatment with linaclotide. We then compared response and side effects reported in patients who required low versus high-dose linaclotide (Table 3). In our cohort, 13 patients (42%, 13/31) were on a high dose prescription whereas 18 patients were on a low-dose regimen (58%, 18/31). Interestingly, diarrhea, cramping and bloating were more commonly identified in the low-dose group, with 50% (9/18) in low dose and 15% (2/13) in the low dose group (*p* = 0.066) reporting these symptoms. Six patients (1 low dose and 5 high dose) stopped the medication due to lack of efficacy with this treatment. This ranged from short term failures (3 months) to discontinuation after 51 months of usage.

**Table 3:** Linaclotide usage

Clinical variable	High-dose Linaclotide (n = 13)	Low-dose Linaclotide (n = 18)	p-value
Frequency, doses per week, median (IQR)	7 (7–7)	7 (1–7)	0.774
Average daily dose in mcg, mean (SD)	273 (32)	137 (24)	< 0.0001
Side effect			
Abdominal pain, % (n)	15 (2/13)	11 (2/18)	1.0000
Diarrhea, cramping, or bloating, % (n)	15 (2/13)	50 (9/18)	0.0656
Nausea, % (n)	8 (1/13)	6 (1/18)	1.0000
Unspecified/did not tolerate, % (n)	8 (1/13)	6 (1/18)	1.0000
Length of treatment in months (SD)	21 (12–50)	13.5 (7–27)	0.1382
Weight change on linaclotide, lb, median (IQR)	0.8 (− 3.8 to 1.4)	− 0.6 (− 3.7 to 1.4)	0.5914

High-dose: > 145 mcg daily linaclotide, low-dose: ≤ 145 mcg daily

*IQR* intra-quartile range, *SD* standard deviation

## Discussion

This is the first report examining the tolerability and clinical response of SSc patients with severe GI disease treated with linaclotide for constipation. Our experience suggests that linaclotide is a safe and effective option in SSc patients with significant lower GI disease manifestations who have not responded adequately to other medications. Patients used linaclotide for a mean duration of 22.6 months for treatment. We found that 90% (28/31) of patients had a favorable response to linaclotide, either as monotherapy or when used in combination with other agents. While, diarrhea, bloating, and abdominal pain were common side effects, no major drug-related adverse events were reported in this cohort of SSc patients, which was likely related to the minimal systemic absorption of this medication. Guidelines for electrolyte monitoring have not been developed for this patient population, though the European Medicines Agency (EMA) recommend monitoring of patients “prone to a disturbance of water or electrolyte balance should be specifically monitored” [15].

The majority of patients in this study had severe lower GI disease, with 74% having a history of recurrent pseudo-obstruction or malabsorption (n = 20) and/or dependence on total parenteral nutrition (n = 3). Though pseudo-obstruction in SSc has historically been attributed to small bowel dysmotility, in our Scleroderma Center, we have found that pseudo-obstruction in SSc is instead associated with severe colonic hypomotility [16]. A recent study also determined that severe colonic involvement is under-reported in the SSc population, and that this complication is associated with a high mortality rate of 27% [4]. Table 1 illustrates that the patients in our cohort tried multiple medications without sufficient symptomatic relief prior to linaclotide initiation. Many of these medications were continued or added for further optimization after linaclotide initiation, highlighting that linaclotide can be effective as monotherapy or in combination with other agents for constipation in SSc. Linaclotide now provides physicians with another therapeutic option for the management of such patients in the clinical setting. Prospective randomized-controlled studies will be important in determining whether the early initiation of linaclotide in patients with lower GI disease manifestations, such as colonic dysmotility, can reduce the risk of recurrent pseudo-obstruction, hospitalization, and death in the longer term.

The majority of patients in our study (90%) on linaclotide therapy reported an improvement in their symptoms. Two patients discontinued therapy due to a lack of efficacy of high-dose linaclotide, and these patients had also not responded to lubiprostone, pyridostigmine, or prucalopride. Importantly, one of these non-responders was also on opiate medications (124 morphine equivalent dosing/day), suggesting that rheumatologists should consider specifically targeting opioid-induced constipation when appropriate (i.e. methylnaltrexone).

We had objective GI transit data from thirteen patients in the cohort who underwent nuclear medicine-based whole gut scintigraphy, and 76.9% (10/13) of patients had significantly delayed colonic transit at 72 h. Despite normal transit by scintigraphy in three patients, all had relief with linaclotide, suggesting that SSc patients may benefit from linaclotide whether or not they have objective evidence of dysmotility. This may be related to the dual action of linaclotide, as both a pro-secretory and pro-kinetic agent. Among the patients with significant lower bowel symptoms and normal colonic transit, none of these patients required high-dose linaclotide. Importantly, only one patient had evidence of small bowel dysmotility on whole gut scintigraphy, showing that this was not a major cause of dysfunction in our patients. Two of the three patients with normal whole gut scintigraphy did have abnormal anorectal manometry highlighting that SSc patients may have other mechanisms for constipation (e.g. anorectal dysfunction) outside of transit delays [4, 17]. Characterizing important GI subsets and targeting therapy within the SSc population may help optimize GI treatment responses.

Our study has many strengths. We report a large case series of patients with SSc who were treated with linaclotide for refractory constipation. All patients were seen in the Johns Hopkins Scleroderma Center with standardized data collection and medication reconciliation. The patients in our study have a long follow-up time and systematic screening of a spectrum of SSc-specific clinical features and autoantibodies. As a retrospective series, our data collection was limited by the intrinsic heterogeneity related to clinical documentation, the absence of a matched control group, and reliance on patient-reported compliance. Additionally, given that lower GI manifestations of systemic scleroderma is uncommon, we did not have the ability to power our analysis to identify patients with the best responses to therapy. Addressing these concerns will be a focus of future work.

## Conclusions

Linaclotide may be a safe and effective treatment for SSc patients with significant lower GI disease who do not respond to standard treatments. We find that despite severe disease, many patients experienced relief with low-dose linaclotide. Determining whether early initiation of linaclotide could reduce or prevent pseudo-obstruction or other severe complications in patients at risk will be an important question for future studies.

## Data Availability

The datasets during and/or anaylsed during the current study available from the corresponding author on reasonable request.

## Abbreviations

GI: Gastrointestinal
SSc: Systemic sclerosis
GC-C: Guanylate cyclase-C
cGMP: Cyclic guanosine monophosphate
ACR: American College of Rheumatology
UCLA GIT: University of California, Los Angeles Gastrointestinal Tract Instrument
EMA: European Medicines Agency
SD: Standard deviation
IQR: Interquartile range
RP: Raynaud’s phenomenon
Mcg: Microgram

## References

[1] Gyger G, Baron M (2012). Gastrointestinal manifestations of SSc: recent progress in evaluation, pathogenesis, and management. Curr Rheumatol Rep.

[2] Steen VD, Medsger TA (2000). Severe organ involvement in systemic sclerosis with diffuse scleroderma. Arthritis Rheum.

[3] Shreiner AB, Murray C, Denton C (2016). Gastrointestinal manifestations of systemic sclerosis. J Scleroderma Relat Disord.

[4] Brandler JB, Sweetser S, Khoshbin K (2019). Colonic manifestations and complications are relatively under-reported in systemic sclerosis: a systematic review. Am J Gastroenterol.

[5] Nagaraja V, McMahan ZH, Getzug T (2015). Management of gastrointestinal involvement in scleroderma. Curr Treatm Opt Rheumatol.

[6] Murtaugh MA, Frech TM (2013). Nutritional status and gastrointestinal symptoms in systemic sclerosis patients. Clin Nutr.

[7] Brierley SM (2012). Guanylate cyclase-C receptor activation: unexpected biology. Curr Opin Pharmacol.

[8] Castro J, Harrington AM, Hughes PA (2013). Linaclotide inhibits colonic nociceptors and relieves abdominal pain via guanylate cyclase-C and extracellular cyclic guanoside 3’,5’-monophosphate. Gastroenterology.

[9] Rao S, Lembo AJ, Shiff SJ (2012). A 12-week randomized, controlled trial with a 4-week randomized withdrawal period to evaluate the efficacy and safety of linaclotide in irritable bowel syndrome with constipation. Am J Gastroenterol.

[10] Chey WD, Lembo AJ, Lavins BJ (2012). Linaclotide for irritable bowel syndrome with constipation: a 26-week, randomized double-blind, placebo-controlled trial to evaluate efficacy and safety. Am J Gastroenterol.

[11] Farmer AD, Ruffle JK, Hobson AR (2019). Linaclotide increases cecal pH, accelerates colonic transit, and increases colonic motility in irritable bowel syndrome with constipation. Neurogastroenterol Motil.

[12] Bassotti G, Battaglia E, Bachetti F (2019). Long-term treatment with linaclotide of intestinal pseudo-obstruction secondary to Ehlers-Danlos syndrome. Dig Liver Dis.

[13] Van den Hoogen F, Khanna D, Fransen J (2013). 2013 classification criteria for systemic sclerosis: an American College of Rheumatology/European League Against Rheumatism collaborative initiative. Arthritis Rheum.

[14] Khanna D, Hays RD, Maranian P (2009). Reliability and validity of the University of California, Los Angeles Scleroderma Clinical Trial Consortium Gastrointestinal Tract Instrument. Arthritis Rheum.

[15] Berntgen M, Enzmann H, Schabel E (2013). Linaclotide for treatment of irritable bowel syndrome: the view of European regulators. Dig Liver Dis.

[16] McMahan Z, Tucker AE, Perin J, et al. The relationship between gastrointestinal transit, Medsger GI severity, and UCLA GIT 2.0 symptoms in patients with systemic sclerosis. *Arthritis Care Res (Hoboken)*. 2020. Epub ahead of print. 10.1002/acr.24488 PMC8050123 33064934

[17] Cao H, Liu X, An Y (2017). Dysbiosis contributes to chronic constipation development via regulation of serotonin transporter in the intestine. Sci Rep.

